# Emergence and Characterization of Acute Coronary Syndrome in Adults After Confirmed or Missed History of Kawasaki Disease in Japan: A Japanese Nationwide Survey

**DOI:** 10.3389/fped.2019.00275

**Published:** 2019-07-09

**Authors:** Yoshihide Mitani, Etsuko Tsuda, Hitoshi Kato, Takashi Higaki, Masako Fujiwara, Shunichi Ogawa, Fumiko Satoh, Yoshikazu Nakamura, Kei Takahashi, Mamoru Ayusawa, Tohru Kobayashi, Fukiko Ichida, Masaki Matsushima, Masahiro Kamada, Kenji Suda, Hiroyuki Ohashi, Hirofumi Sawada, Takaaki Komatsu, Kenji Waki, Masanori Shinoda, Ryusuke Tsunoda, Hiroyoshi Yokoi, Kenji Hamaoka

**Affiliations:** ^1^Department of Pediatrics, Mie University Graduate School of Medicine, Tsu, Japan; ^2^Department of Pediatric Cardiology, National Cerebral and Cardiovascular Center, Suita, Japan; ^3^Division of Clinical Research Planning, Department of Development Strategy and Cardiology, Center for Clinical Research and Development, National Center for Child Health and Development, Tokyo, Japan; ^4^Department of Regional Pediatrics and Perinatology, Ehime University Graduate School of Medicine, Toon, Japan; ^5^Department of Pediatrics, Jikei University School of Medicine, Tokyo, Japan; ^6^Department of Pediatrics, Nippon Medical School, Tokyo, Japan; ^7^Department of Forensic Medicine, Tokai University School of Medicine, Isehara, Japan; ^8^Department of Public Health, Jichi Medical University, Shimotsuke, Japan; ^9^Department of Pathology, Toho University Medical Center, Ohashi Hospital, Tokyo, Japan; ^10^Department of Pediatrics and Child Health, Nihon University School of Medicine, Tokyo, Japan; ^11^Department of Pediatrics, Toyama University School of Medicine, Toyama, Japan; ^12^Department of Pediatric Cardiology, Chukyo Hospital, Nagoya, Japan; ^13^Department of Pediatric Cardiology, Hiroshima City Hiroshima Citizens Hospital, Hiroshima, Japan; ^14^Department of Pediatrics and Child Health, Kurume University, Kurume, Japan; ^15^Department of Cardiology, Dokkyo Medical University Koshigaya Hospital, Koshigaya, Japan; ^16^Department of Pediatrics, Kurashiki Central Hospital, Kurashiki, Japan; ^17^Department of Cardiology, Toyota Kosei Hospital, Toyota, Japan; ^18^Department of Cardiology, Japanese Red Cross Kumamoto Hospital, Kumamoto, Japan; ^19^Cardiovascular Center, Fukuoka Sanno Hospital, Fukuoka, Japan; ^20^Department of Pediatric Cardiology and Nephrology, Graduate School of Medical Science, Kyoto Prefectural University of Medicine, Kyoto, Japan

**Keywords:** Kawasaki disease, coronary aneurysm, acute coronary syndrome, transition, long-term issue

## Abstract

**Background:** Acute coronary syndrome (ACS), which is emerging in adults long after confirmed (followed-up or lost-to-follow), or missed Kawasaki disease (KD), is poorly characterized.

**Methods and Results:** A Japanese retrospective nationwide hospital-based questionnaire survey of ACS during 2000–09 was conducted to characterize such patients. Among a total of 67 patients (median age 35, male 76%) recruited, low conventional coronary risks (≤1/6) was noted in 75%, a diagnosis of ST-elevation and myocardial infarction or cardiac arrest in 66%, medication before ACS in 22% (warfarin in 4%), and no prior history of acute myocardial infarction in 94%. One-month mortality was 19%. KD diagnosis was made in 32 during acute illness (Group A), in which 17 were lost to follow, and retrospectively in the other 35 from coronary imaging at ACS (Group B). Group A developed ACS at lower coronary risks (≤2/5 in 87 vs. 65% in group B, *p* = 0.043) at a younger age (26.5 vs. 40 yo, *p* < 0.001). In group A, followed-up patients developed ACS under medication before ACS (87 vs. 0% in lost-to-follow patients, *p* < 0.001) for giant aneurysm in culprit lesions (69 vs. 29%, *p* = 0.030). One-month mortality was comparable between groups A and B, and between patients followed-up and lost-to-follow in group A. The culprit lesion in group A was characterized by the association of an aneurysm ≥6 mm in acute KD (100%), lack of significant stenosis (61%) or giant aneurysm (50%) in the long-term (median interval 16 y), and the presence of intravascular ultrasound-derived calcification at ACS (86%).

**Conclusions:** The present retrospective nationwide questionnaire survey demonstrated nationwide emergence of initial ACS in young adults at low coronary risks, who are followed-up or lost-to-follow after confirmed KD and initial coronary aneurysms ≥6 mm.

## Introduction

Kawasaki disease (KD) is a common acute febrile disorder of unknown etiology in young children, which is associated with systemic vasculitis especially in coronary circulation (1, 2). Previously, up to 25% of KD patients develop coronary aneurysms leading to lethal coronary vascular events, which occurs typically early after the acute illness in infants associated with giant aneurysms (1, 2). Recently, this disease is a great concern to cardiologists as well as pediatricians, because grown-up KD patients with coronary sequelae exhibit functional and structural alterations in the coronary arteries (3–7). Such patients, in fact, seem to have a coronary event long after the acute illness in adulthood, which is poorly characterized (8–12).

It has been 50 years since the initial report of KD was published in 1967, with the first English version of the same report in 1974 (13, 14). As of Dec 2010, >272,000 patients were affected by KD in Japan, among whom >117,000 reached adulthood; it is estimated that there are **>**24,000 young adults with a history of KD in the United States (9, 15). Reports of acute coronary events in young adults after a missed history of KD began to emerge as early as in 1980s after Kawasaki's original publication (8, 10, 16, 17). Recently, case review reports showed that young adults mainly long after missed KD developed acute events mimicking acute coronary syndrome (ACS), which includes acute myocardial infarction (AMI), unstable angina and sudden cardiac arrest (10, 17, 18). However, a significant knowledge gap exists between the onset of acute KD, the initial coronary involvement and the occurrence of KD-associated ACS in adulthood. Limitations in retrospective diagnosis of missed KD and the lack of recognition of coronary involvement even in patients with a confirmed KD diagnosis in early decades, which may have resulted in loss of follow-up, hampered the investigation of this condition (8, 10, 19). In fact, after Kato's study on adult population with a missed history of KD in 1992, no subsequent survey from Japan has been reported to characterize this condition (16).

Recently in Japan, there was a 3.5-fold increase in the adult population with registered KD diagnosis during the acute illness from 1998 to 2010 (15, 20). It is >20 years, as of the year 2000, after the first diagnostic criteria of KD was released for the first nationwide survey of acute KD in 1970 and the first angiographic and ultrasound findings of coronary artery lesions after KD were reported in 1975 and1979, respectively (21–25). Accordingly, a substantial population with registered KD diagnosis and coronary sequelae should have begun to reach adulthood in 2000s in Japan. We therefore conducted a Japanese nationwide survey to characterize ACS, which is emerging in adults with a confirmed (whether followed-up or lost to follow-up) as well as missed history of KD.

## Methods

### Study Design

This was a retrospective, nationwide hospital-based questionnaire survey of ACS occurring during 2000–2009 in adults (≧ 20 years of age) after a confirmed or missed KD in Japan. This was an official research project, which was endorsed by the Japanese Society of Kawasaki Disease and was conducted by members in the subcommittee on ACS in adult KD patients in the Society. The ethics committee in the Mie University Graduate School of Medicine approved this study. A confirmed diagnosis of KD was defined as the KD diagnosis at the acute illness by medical doctors, which was verified in accordance with medical documents (medical record or medical referral letter) or interview with the patients or family members about KD diagnosis by medical doctors during the acute illness (24, 26, 27). A missed KD diagnosis was defined as the KD diagnosis at ACS or other time points, according to findings in coronary imaging, including coronary aneurysm, ring-like calcification, recanalization, or localized severe intimal thickening, or autopsy findings, not by KD-like symptoms retrospectively obtained from interviews with parents (26, 27). Study setting was described in detail in the Supplemental Material.

### Data Collection

Questionnaires were sent to the directors in the department of pediatric cardiology or cardiology in 644 hospitals across Japan during 2010–2011 (28, 29), including (1) all hospitals registered as teaching hospitals by the Japanese Society for Pediatric Cardiology and Cardiac Surgery or to which any councilor of the society belonged, (2) all hospitals, from which any doctor in any scientific meeting reported web-searchable cases that met the criteria for inclusion, and (3) all hospitals, in which ≥200 coronary interventional procedures were performed in 2009. Registry data were collected retrospectively by doctors in charge of the patients in each hospital. Inclusion criteria was an adult patient (≥20 years of age) with acute coronary events consistent with ACS, including acute myocardial infarction, unstable angina and sudden cardiac arrest during January 2000-December 2009 (30, 31), the final diagnosis with ACS made clinically with the use of any investigations, at least including coronary angiography or autopsy (30, 31), and the presence of prospective or retrospective diagnosis of KD by medical doctors. Exclusion criteria included any cases diagnosed finally with effort angina, other cardiovascular disorders including arrhythmia, cases for elective coronary interventions, or acute coronary events without diagnosis using coronary angiography or autopsy. Duplication of the reported cases was avoided by investigators through carefully checking the demography (the birthdate and gender), KD diagnosis at acute illness (age and calendar year) and various clinical information at ACS onset, including age, calendar year, the address of the hospital, angiographic findings and follow-up information. Detailed questionnaire forms were shown in the Supplemental Material.

### Statistical Analysis

All statistical analyses were performed with IBM SPSS statistics, version 22. Continuous data are reported as median and interquartile ranges. The significance of any differences among two groups was assessed by the Mann-Whitney U test. Categorical data are expressed as a value or frequency of occurrence. The difference of the proportions of categorical variables among groups was assessed by chi-square analysis. All tests were 2-tailed, and *P* < 0.05 was considered to indicate statistical significance. YM has full access to all the data in the study and takes responsibility for its integrity and the data analysis.

## Results

**Figure 1:**
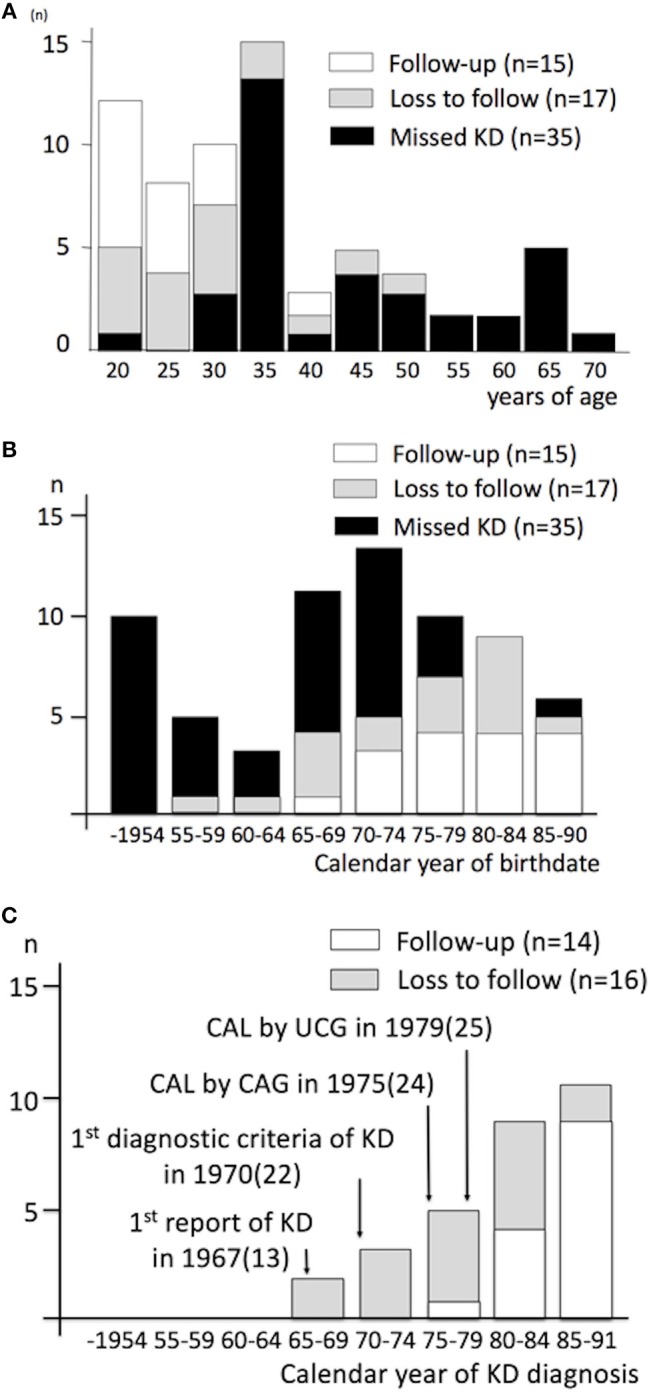
Age at ACS and calendar year of birthdate and KD diagnosis, by the type of KD diagnosis and the follow-up status. **(A)** Age at acute coronary syndrome, **(B)** Calendar year of birthdate, **(C)** Calendar year of KD diagnosis. ACS denotes acute coronary syndrome; KD, Kawasaki disease; CAL, coronary artery lesion; CAG, coronary angiography; UCG, ultrasound cardiography; n, number of patients. Number in parenthesis indicates reference number.

The primary response rate in the present survey was 46.3%. A total of 67 patients (median age 35, male 76%) were recruited (Table 1). A confirmed diagnosis of KD was made by medical doctors in 32 during acute illness (Group A, verified by medical documents in 22; obtained by interview with family members in 10), among which 17 were lost to follow up at ACS. A missed KD diagnosis was made retrospectively in the other 35 patients in accordance with coronary imaging (*n* = 35) (*n* = 34 at the registered ACS events, *n* = 1 at previous AMI in adulthood) and autopsy (*n* = 0) at ACS (Group B), in which episodes suggestive of acute KD during childhood were also obtained in the parent interviews for 2 patients. Demography and clinical characteristics by the group were shown (Table 1; Figure 1). All the patients, except one Korean and two of unknown ethnicity in group B, were Japanese. Among overall patients, low conventional coronary risks (≤1/6) was noted in 75%, a diagnosis of ST-elevation and myocardial infarction (STEMI) or cardiac arrest in 66%, chest pain in 85%, exercise-related events in 24%, medication before ACS in 22% (warfarin in 4%), and no prior history of AMI in 94%. One-month mortality was 19%. Compared with group B, group A was characterized by younger age at ACS (26.5 vs. 40 yo, *p* < 0.001), a more recent birthdate (birth in 1965 or after, 94 s. 54%, < 0.001), lower conventional coronary risks (≤1/6 in 87 s. 65%, *p* = 0.043) (smoking, 30 vs. 53%, *p* = 0.064) and higher percentage of medication before ACS (41 vs. 6%, *p* = 0.001). In group A, patients who were followed up before ACS were characterized by a more recent onset (1980 and after) of the acute illness (93 vs. 44% in KD patients lost to follow up, *p* = 0.004), higher percentage of medication (87 vs. 0%, *p* < 0.001) before ACS, and a tendency to have a history of percutaneous coronary intervention (PCI) before registered ACS (20 vs. 0%, *P* = 0.053). One-month mortality was comparable between Groups A and B, and between patients followed-up and lost to follow in group A (Figure 1; Table 1).

**Table 1:** Clinical characteristics.

	**Total**	**KD diagnosis during acute illness**			***p***	**KD diagnosis at ACS event**	***p***
		**Follow**	**Lost to follow**	**Total**			
*N*	67	15	17	32		35	
Median age at ACS					0.104		<0.001
yo	35	25	30	26.5		40	
(quartiles)	(26–47)	(21–30)	(24.5–38.5)	(23.5–33)		(36–57)	
Male gender, *n* (%)	51 (76)	11 (73)	16 (94)	27 (84)	0.106	24 (69)	0.130
Median age at acute illness, /*N*		/14	/16	/30	0.208		
yo (quartiles)		4 (1–8)	3 (1–3.5)	3 (1–5)			
**Calendar year at birthdate**							
Year 1965 and after, *n* (%)	48 (72)	15 (100)	15 (88)	30 (94)	0.170	19 (54)	<0.001
Calendar year at acute illness		/14	/16	/30	0.004		
Year 1980 and after, *n* (%)		13 (93)	7 (44)	20 (67)			
Conventional risk factor, /*N*	/64	/14	/16	/30		/34	
Smoking	27 (42)	3 (21)	6 (38)	9 (30)	0.338	18 (53)	0.064
0–1, *n* (%)	48 (75)	12 (86)	14 (88)	26 (87)	0.886	22 (65)	0.043
0	23	11	8	19		4	
1	25	1	6	7		18	
2	11	2	2	4		7	
3	5	0	0	0		5	
**ACS type**, ***n*** **(%)**							
STEMI/Cardiac arrest	44 (66)	11 (73)	9 (53)	20 (63)	0.234	24 (69)	0.601
STEMI	35 (52)	8	7	15		20	
Cardiac arrest	9 (13)	3	2	5		4	
Unstable angina	17 (25)	3	6	9		8	
Non-STEMI	6 (9)	1	2	3		3	
**Symptom at ACS**							
Chest pain, *n*(%)	57 (85)	11 (73)	14 (82)	25 (78)	0.538	32 (91)	0.127
Shock, *n*	13	2	3	5		8	
Physical status at ACS, /*N*	/63	/13	/15	/28		/35	
Exercise-related, *n* (%)	15 (24)	5 (38)	3 (20)	8 (29)	0.281	7(20)	0.427
**Exercise-unrelated**							
At rest	38	6	10	16		22	
During sleeping	10	2	2	4		6	
**Medication before ACS**							
Medication (+), *n*(%)	15 (22)	13 (87)	0 (0)	13(41)	<0.001	2 (6)	0.001
Antiplatelet	15	13	0	13		2	
Warfarin	3 (4)	3 (20)	0 (0)	3 (9)		0 (0)	
β blocker	8	6	0	6		2	
Nitrate	2	2	0	2		0	
ACEI/ARB	3	1	0	1		2	
Statin	0	0	0	0		0	
PCI before ACS, *n* (%)	4 (6)	3 (20)	0 (0)	3 (9)	0.053	1 (3)	0.261
CABG before ACS, *n* (%)	2 (3)	0 (0)	1 (6)	1 (3)	0.340	1 (3)	0.949
History of AMI, *n* (%)	4 (6)	1 (7)	1 (6)	2 (6)	0.927	2 (6)	0.926
Medication 1m after ACS, /*N*	/52	/10	/14	/24		/28	
Medication (+), *n*(%)	51 (98)	10 (100)	13 (93)	23 (96)	0.388	28 (100)	0.275
Antiplatelet	51	10	13	23		28	
Warfarin	29 (56)	7 (70)	6 (43)	13 (54)		16 (57)	
β blocker	18	4	1	15		13	
Nitrate	8	2	3	5		3	
ACEI/ARB	23	3	3	6		17	
Statin	18	0	6	6		12	
Death at one month, *n* (%)	13 (19)	4 (27)	3 (18)	7 (22)	0.538	6 (17)	0.625

*KD, denotes Kawasaki disease; ACS, acute coronary syndrome; STEMI, ST elevation myocardial infarction; ACEI, angiotensin converting enzyme inhibitor; ARB, angiotensin II receptor blocker; PCI, percutaneous coronary intervention; CABG, coronary artery bypass grafting; AMI, acute myocardial infarction. Traditional coronary factors included dyslipidemia, hypertension, smoking, diabetes, and family history of AMI. If missing data was included in any item, the number of available data was specified as /N. Percentages, median values and quartile ranges were calculated on the basis of the available data in each item*.

Of the culprit lesions, 67 patients were characterized by demonstrable thrombosis (74%), as well as the presence of persistent giant aneurysms (≥8 mm) (38%) and intravascular ultrasound (IVUS)-derived calcification (92%) (Table 2). In group A, patients who were followed up before ACS represented a higher proportion of persistent giant aneurysms in culprit lesions (69 vs. 29%, *p* = 0.030) than those lost to follow. In group A, 28% received intravenous immunoglobulin (IVIG) treatment during the acute illness (Table 3). The vessel size of prospective culprit lesion was 6.0–7.9 mm in 36% and ≥8 mm in 64%, and patients with multi-vessel coronary involvement accounted for 71% in the convalescence of acute KD. Prospective culprit lesions were associated with significant stenosis in 39% and giant aneurysm in 50% in the long-term follow-up period (16 y, 13–18 y, after the acute illness).

**Table 2:** Characteristics of the culprit lesions.

	**Total**	**KD diagnosis during acute illness**			**KD diagnosis**		
		**Follow**	**Lost to follow**	**Total**	***p***	**At ACS event**	***p***
Number of patients	67	15	17	32		35	
Culprit lesion, /*N*	/64	/13	/16	/29		/35	
RCA, *n* (%)	32 (50)	6 (46)	8 (50)	14 (48)		18 (51)	
LCA, *n* (%)	35 (55)	7 (55)	8 (50)	15 (52)	0.837	20 (57)	0.665
LMT, *n*	6	1	1			3	
LAD, *n*	28	7	6			15	
LCX, *n*	8	2	2			5	
Demonstrable thrombosis, /*N*	/57	/9	/16	/25	0.282	/32	0.142
*n* (%)	42(74)	7(78)	9 (56)	16 (64)		26(81)	
Size of AN in the vicinity, /*N*	/61	/13	/17	/30		/31	
3.0–3.9 mm	11	1	4	5		6	
4.0–5.9 mm	11	1	3	4		7	
6.0–7.9 mm	16	2	5	7		9	
≥8.0 mm	23 (38)	9 (69)	5 (29)	14 (47)	0.030	9 (29)	0.155
IVUS-derived calcification, /*N*	/13	/3	/4	/7	0.350	/6	0.335
*n* (%)	12 (92)	3 (100)	3 (75)	6 (86)		6 (100)	
Emergency treatment, /*N*	/64	/13	/17	/30		/34	
Thrombolysis/Aspiration	29 (45)	6 (46)	4 (24)	10 (33)	0.193	19 (56)	0.071
IC thrombolysis	13	4	3			6	
Thrombus aspiration	23	4	2			17	
POBA	22	4	5			13	
Stenting	18	3	2			13	
CABG	10	2	2			6	

*RCA, denotes right coronary artery; LCA, left coronary artery; LMT, left main coronary trunk; LAD, left anterior descending coronary artery; LCX, left circumflex coronary artery; AN, aneurysm; IVUS, intravascular ultrasound; IC, intracoronary; POBA, plain old balloon angioplasty; CABG, coronary artery bypass grafting*.

**Table 3:** Treatment for acute KD and culprit lesions early and ≥5 years after acute KD.

Treatment for acute KD, /*N*	/18
No specific treatment, *n* (%)	3 (17)
Anti-platelet agent, *n* (%)	14 (78)
Intravenous immunoglobulin, *n* (%)	5 (28)
Steroid, *n* (%)	4 (22)
**CAL during or in the convalescence of acute KD**	
Prospective culprit lesion, /*N*	/14
3.0–3.9 mm, *n* (%)	0 (0)
4.0–5.9 mm, *n* (%)	0 (0)
6.0–7.9 mm, *n* (%)	5 (36)
≥8.0 mm, *n* (%)	9 (64)
Patients with multi-vessel CAL, /N	/14
*n* (%)	10 (71)
**CAL in the long-term (a most recent time point ≥5 years after KD)**	
(median, interquartile range)	16 y (13–18 y)
The prospective culprit lesion, /*N*	/18
≥8 mm AN/stenosis (>75%), *n* (%)	4 (22)
≥8 mm AN/stenosis (≤75%), *n* (%)	5 (28)
<8 mm AN/stenosis (>75%), *n* (%)	3 (17)
<8 mm AN/stenosis (≤75%), *n* (%)	6 (33)

*CAL denotes coronary artery lesion.Number of available patients in each subgroup in ‘Treatment for acute KD': followed-up patients, n = 11; lost-to-follow patients, n = 7. ‘CAL during or in the convalescence of acute KD': followed-up patients, n = 10; lost-to-follow patients, n = 4. ‘CAL ≥5 years after KD': followed-up patients, n = 12; lost-to-follow patients, n = 6*.

## Discussion

The present Japanese nationwide survey demonstrated that ACS in young adults, with a confirmed as well as a missed history of KD, is emerging in Japan. Patients after confirmed KD usually had initial events long after acute KD; culprit lesions were characterized by large or giant AN in the convalescence of acute KD, the lack of giant aneurysms or severe stenosis in more than a half of patients in the long-term, and IVUS-derived calcification at ACS. Compared with lost-to-follow patients, followed-up patients were more likely to have persistent giant aneurysm in the culprit lesions and had an initial ACS even under conventional medication. Compared with patients after confirmed KD, adults after missed KD also had an initial ACS at higher coronary risks at more advanced age. The present findings may give an insight into the natural history of coronary sequelae as well as the emerging ACS in patients after KD in adulthood.

The present study uniquely characterized young adult population who usually developed an initial ACS long after a confirmed diagnosis of KD and initial coronary sequelae in a nationwide manner in Japan. Recruitment of patients with a confirmed KD diagnosis and coronary sequelae in the convalescence of the acute illness in the present study complied with the chronological order of events in KD research in Japan, including the reports of the first diagnostic criteria of KD and the first angiographic and ultrasound findings of coronary artery lesions after KD (Figure 1C) (21, 22, 24, 25). Present findings in confirmed KD cases may not be confounded by the lack of coronary risks, as in missed KD cases in previous literature (8, 10, 11, 17). Correlation of coronary sequelae in the convalescence of acute KD to the culprit lesion at ACS and the absence of patients with normal coronary arteries at the acute KD in group A underlines the contribution of initial coronary sequelae to this condition. ACS in patients lost to follow or ACS in patients after missed KD were not necessarily associated with persistent giant aneurysm in the present study, in contrast to previous reports in adults (10). Such difference in the former may be related to better recognition of KD patients, which is explained by the emergence of population with KD diagnosis during the acute illness in the present study (18); the difference in the latter may be related to the publication bias in the literature, including inclusion criteria of patients (10).

Initial occurrence of ACS in patients with persistent giant aneurysm who were followed up under medication and/or PCI may indicate that such severe cases might become resistant to the conventional anti-thrombotic therapy in adulthood. The gap between ACS events in followed-up patients with persistent giant aneurysms in the present study and the uneventful outcome in long-term pediatric cohort studies could be explained by the larger background population and the longer time interval until adulthood in the present study, as well as the expertise in KD centers in previous studies (32). Conventional coronary risks or the aging may have additional roles in the development of ACS, because patients after missed history of KD had an initial ACS and more coronary risks at a more advanced age (10). These findings are collectively in line with the functional and/or morphological abnormality in the coronary vessel wall in the development of adult ACS (3–7, 33). Since IVUS-derived calcification in the culprit lesions, a parameter for vessel wall morphology was in fact demonstrated in any subgroup of KD adult patients at ACS, and vessel wall changes culminating in IVUS-derived calcification may play a role in the development of ACS. The potential mechanisms underlying these findings include ongoing remodeling of the coronary arteries mediated by endothelial dysfunction, inflammatory process, myofibroblast proliferation and matrix remodeling of the coronary arteries, which is subjected to progressive calcifications located at the subendothelial surface of any sequelae in the long term (6, 33–35). In addition, potentially superimposed atherogenesis might play a role in a subset of patients with coronary aneurysms long after KD, especially in group B, which requires further research (6, 36, 37).

### Implications

Firstly, although the population in which KD-related coronary artery lesion was evaluated during the acute illness in or after 1980 is limited (Figure 1C), the lack of KD patients with normal coronary arteries from the disease onset in the present study may reassure such patients in their 20 s. Secondly, the current AHA and Japanese guidelines recommend anti-platelet therapy for persistent coronary aneurysms or stenosis, but not necessarily for regressed aneurysms (1, 27). Since patients with regressed aneurysms in fact account for a half of the entire KD population with coronary sequelae and may rarely have ACS in early adulthood (2), how to stratify patients at risk is important in an evidence-based approach for this subgroup. Since IVUS-derived coronary calcification was detected in a majority of ACS patients investigated, the present findings suggest that KD patients with regressed aneurysms, especially in case of the original aneurysm ≥6 mm, may be screened by plain MDCT for calcification in adulthood (38). Thirdly, although the presence of persistent giant aneurysms or induced myocardial ischemia has been regarded as an important indicator for poor prognosis in KD, the lack of persistent giant aneurysms or severe stenosis in more than a half of patients in the long-term, in the present study, may uniquely alert cardiologists to the risk of ACS in adulthood (1, 9, 27, 39). Fourthly, since group B is characterized by increased conventional coronary risks and a tendency for an increase in smoking at an advanced age, it may be reasonable to recommend life style modification in KD patients in adulthood (10). Together with the vessel wall alterations in coronary arteries in the long term and initial ACS in followed-up patients under conventional medication, any additional compounds with anti-inflammatory property, such as statins, may be of theoretical benefit in KD patients who are at risk in adulthood (40).

### Limitations

Firstly, there are issues (including recall bias and potential duplication of patients) related to the retrospective nature of the study, as well as issues of a low response rate (46.3%) and missing data, especially during the acute illness and convalescence in this nationwide questionnaire survey. Prospective long-term registry from the KD onset would be required to uncover the natural history of this disorder in adulthood. However, considering the rarity of the events, the long observation period required, and the issue of loss-to-follow in adult KD, a nationwide retrospective survey as in the present study may play a role in characterizing this condition. Secondly, the proportion of regressed aneurysms in culprit lesions at ACS might be undervalued in the loss-to-follow patients after KD or in patients after missed KD in adulthood. A cohort of adult KD population, if any, including patients with regressed aneurysms will be warranted in this regard. Thirdly, KD diagnosis from the coronary imaging at ACS is uncertain in group B and could be biased by the presence of giant aneurysm or the lack of multiple coronary risks. However, this group in this study may be of clinical relevance, because this group exhibits characteristics consistent with the other subgroup: this group and lost-to-follow patients in group A had a reasonably similar proportion of persistent giant aneurysm at ACS; this group is still at more coronary risks than group A. Fourthly, as with all retrospective questionnaire surveys, the integrity and validity of the data are potential limitations of our study.

## Conclusions

The present Japanese retrospective nationwide questionnaire survey uniquely characterized emerging ACS in young adults with a confirmed as well as missed diagnosis of KD, which complies with the chronology of KD research for half a century in Japan. The present findings may alert pediatricians and general practitioners to the risk of ACS in adults long after the acute illness and the transition issues of KD, and warrant a prospective registry study of adults with a confirmed KD diagnosis and the information of initial coronary sequelae.

## Data Availability

The datasets for this manuscript are not publicly available because it is not approved by the ethics committee in Mie University Graduate School of Medicine. Requests to access the datasets should be directed to ymitani@clin.medic.mie-u.ac.jp.

## Ethics Statement

The study conformed to the principles of the Helsinki Declaration, and the study protocol was approved by the Ethics Committee of the Mie University Graduate School of Medicine. The requirement for individual informed consent was waived.

## Author Contributions

YM, HK, KH, and HY design, methodology, investigation, supervision, funding acquisition, data curation, formal analysis, resources, writing, final approval of the manuscript. ET design, methodology, investigation, supervision, acquisition, data curation, formal analysis, resources, writing, final approval of the manuscript. TH, SO, KT, MA, ToK, FI, and HY design, methodology, investigation, supervision, data curation, formal analysis, resources, writing, final approval of the manuscript. MF design, methodology, investigation, data curation, resources, writing, final approval of the manuscript. FS design, methodology, investigation, acquisition, data curation, formal analysis, resources, writing, final approval of the manuscript. YN design, methodology, investigation, supervision, data curation, formal analysis, writing, final approval of the manuscript. MM, MK, KS, HO, HS, TaK, KW, MS, and RT design, methodology, investigation, data curation, formal analysis, resources, writing, final approval of the manuscript.

### Conflict of Interest Statement

The authors declare that the research was conducted in the absence of any commercial or financial relationships that could be construed as a potential conflict of interest.
