# Supplementary material for: Emergence and Characterization of Acute Coronary Syndrome in Adults After Confirmed or Missed History of Kawasaki Disease in Japan: A Japanese Nationwide Survey
Source: Front Pediatr. 2019 Jul 9;7:275. doi: 10.3389/fped.2019.00275 (PMC6629790; doi:10.3389/fped.2019.00275)
Supplement: Supplementary file 1 [file Table_1.DOCX]

**SUPPLEMENTAL MATERIAL**

**Supplemental methods**

**Study Setting**

After Dr Kawasaki initially reported cases of KD in 1967 in Japanese, the first diagnostic criteria of KD was established and disseminated for the first nationwide survey of acute KD in 1970(Kawasaki, 1967;Ayusawa et al., 2005;Uehara R, 2005;Group, 2014). After the first survey, similar survey has been conducted every two years, in which questionnaires and diagnostic guideline prepared by the Japanese Kawasaki Disease Committee were sent by mail to the chief of Departments of Pediatrics in hospitals equipped with a total of 100 or more beds throughout Japan, as described previously(Yanagawa et al., 1986;Nakamura et al., 1992;Uehara R, 2005;Makino et al., 2015). After a steady increase in the number of registered cases intervened by three nationwide epidemics in 1972, 1982 and 1986, KD has been recognized as a common childhood disease among pediatricians in Japan: in 2012, the annual incidence of KD was 264.8 per 100,000 children aged 0-4 years and the cumulative incidence was 1,004 per 100,000 children aged 5-9 years(Yanagawa et al., 1986;Nakamura et al., 1992;Makino et al., 2015). As the age of registered KD cases advances, the number of adults with a confirmed history of KD increased by 3.5-fold from 1998 to 2010: 33,748/139,581 (24.2%) as of Dec1998 and 117,964/247,690 (47.6%) as of Dec 2010(Yanagawa, 1999;Nakamura Y, 2011). It is >20 years since the coronary artery lesion as sequelae of KD began to be detected by ultrasound or by coronary angiography in 2000s(Kato et al., 1975;Yoshikawa et al., 1979). After the dissemination of the concept of ACS as a unifying thromboembolic syndrome integrating acute myocardial infarction, unstable angina and sudden cardiac arrest around 2000, ACS recognized during the coronary events is emerging in 2000s(Fuster and Kovacic, 2014): the first reported case, in which the diagnosis of ACS was made in a young KD adult with demonstrable acute coronary thrombosis, was experienced in Japan in 2000(Negoro et al., 2003).

**Questionnaire forms**

Questionnaire forms included (1) demography (gender, birthdate), (2) with respect to KD diagnosis during the acute illness, the source of information (medical record or referral letter, interview with the patient or family members); calendar year and age at acute KD; treatment (intravenous immunoglobulin, steroid, antiplatelet agents); presence of multi-vessel aneurysms; size of the aneurysm (3-3.9mm, 4-5.9mm,6-7.9mm, ≥8mm) during the acute illness or the convalescence in the prospective culprit lesions (Newburger et al., 2004;Ogawa, 2011;Group, 2014), (3) with respect to KD diagnosis made retrospectively at ACS, source of information (imaging modalities or autopsy findings); findings in coronary angiography, multi-detector computed tomography (MDCT), IVUS (coronary aneurysm, recanalization, calcification, severe intimal thickening) and pathology(Erbel et al., 1999), (4) clinical characteristics in registered ACS, age; symptoms (chest pain, shock, arrhythmia, fainting, easy fatigability, dyspnea); physical status (during or after exercise, at rest, sleeping); subtypes of ACS (unstable angina, ST-elevation and non-ST elevation myocardial infarction, sudden cardiac arrest) (Krumholz et al., 2008); conventional coronary risk factors (dyslipidemia, hypertension, smoking, diabetes, family history of AMI), follow-up status at ACS (follow-up with or without medication, lost to follow-up) at the time of ACS; medication (anti-platelet agents, warfarin, β blocker, nitrates, angiotensin converting enzyme inhibitor (ACEI)/angiotensin II receptor blocker (ARB), statin), invasive treatments including coronary artery bypass grafting (CABG) and percutaneous coronary intervention (PCI), and myocardial infarction before registered ACS; medication and prognosis (survival) one month after ACS, (5) characteristics of the culprit lesions at ACS: vessels (right coronary artery, left main coronary trunk, left anterior descending and left circumflex coronary artery); thrombus demonstrated by coronary angiograms; size of the aneurysm (3-3.9mm, 4-5.9mm,6-7.9mm, ≥8mm) of which the size was determined by the maximal size of any angiographically visible aneurysm in the vicinity of the culprit lesion during the procedure (Newburger et al., 2004;Ogawa, 2011); obstructive lesion (≤50%, 51-75%, 76-99%, 100%)(Scanlon et al., 1999;Ogawa, 2011) and intravascular ultrasound (IVUS) findings (no plaque, soft, fibrous, calcified, mixed plaque) at the culprit lesion just before, just after, or mid-term after PCI(Erbel et al., 1999); rescue treatment including intravenous or intracoronary thrombolysis, thrombus aspiration, plain old balloon angioplasty (POBA), stenting, and emergency CABG, (6) with respect to prospective culprit lesions before ACS, coronary aneurysms (3-3.9mm, 4-5.9mm,6-7.9mm, ≥8mm) and obstructive lesion (≤50%, 51-75%, 76-99%, 100%) in the prospective culprit lesion by any modalities (MDCT, MRI, CAG) in the mid-term (a most recent time point ≥5 years after KD) (Scanlon et al., 1999;Newburger et al., 2004;Ogawa, 2011;Group, 2014).

**References**

Ayusawa, M., Sonobe, T., Uemura, S., Ogawa, S., Nakamura, Y., Kiyosawa, N., Ishii, M., Harada, K., and Kawasaki Disease Research, C. (2005). Revision of diagnostic guidelines for Kawasaki disease (the 5th revised edition). *Pediatr Int* 47**,** 232-234.

Erbel, R., Ge, J., Gorge, G., Baumgart, D., Haude, M., Jeremias, A., Von Birgelen, C., Jollet, N., and Schwedtmann, J. (1999). Intravascular ultrasound classification of atherosclerotic lesions according to American Heart Association recommendation. *Coron Artery Dis* 10**,** 489-499.

Fuster, V., and Kovacic, J.C. (2014). Acute coronary syndromes: pathology, diagnosis, genetics, prevention, and treatment. *Circ Res* 114**,** 1847-1851.

Group, J.C.S.J.W. (2014). Guidelines for diagnosis and management of cardiovascular sequelae in Kawasaki disease (JCS 2013). Digest version. *Circ J* 78**,** 2521-2562.

Kato, H., Koike, S., Yamamoto, M., Ito, Y., and Yano, E. (1975). Coronary aneurysms in infants and young children with acute febrile mucocutaneous lymph node syndrome. *J Pediatr* 86**,** 892-898.

Kawasaki, T. (1967). [Acute febrile mucocutaneous syndrome with lymphoid involvement with specific desquamation of the fingers and toes in children]. *Arerugi* 16**,** 178-222.

Krumholz, H.M., Anderson, J.L., Bachelder, B.L., Fesmire, F.M., Fihn, S.D., Foody, J.M., Ho, P.M., Kosiborod, M.N., Masoudi, F.A., Nallamothu, B.K., American College of Cardiology/American Heart Association Task Force on Performance, M., American Academy of Family, P., American College of Emergency, P., American Association Of, C., Pulmonary, R., Society for Cardiovascular, A., Interventions, and Society of Hospital, M. (2008). ACC/AHA 2008 performance measures for adults with ST-elevation and non-ST-elevation myocardial infarction: a report of the American College of Cardiology/American Heart Association Task Force on Performance Measures (Writing Committee to develop performance measures for ST-elevation and non-ST-elevation myocardial infarction): developed in collaboration with the American Academy of Family Physicians and the American College of Emergency Physicians: endorsed by the American Association of Cardiovascular and Pulmonary Rehabilitation, Society for Cardiovascular Angiography and Interventions, and Society of Hospital Medicine. *Circulation* 118**,** 2596-2648.

Makino, N., Nakamura, Y., Yashiro, M., Ae, R., Tsuboi, S., Aoyama, Y., Kojo, T., Uehara, R., Kotani, K., and Yanagawa, H. (2015). Descriptive epidemiology of Kawasaki disease in Japan, 2011-2012: from the results of the 22nd nationwide survey. *J Epidemiol* 25**,** 239-245.

Nakamura, Y., Yanagawa, H., and Kawasaki, T. (1992). Mortality among children with Kawasaki disease in Japan. *N Engl J Med* 326**,** 1246-1249.

Nakamura Y, Y.M. (2011). Epidemiology of Fatal cases with Kawasaki disease. *Cardioangiography* 69**,** 412-420.

Negoro, N., Nariyama, J., Nakagawa, A., Katayama, H., Okabe, T., Hazui, H., Yokota, N., Kojima, S., Hoshiga, M., Morita, H., Ishihara, T., and Hanafusa, T. (2003). Successful catheter interventional therapy for acute coronary syndrome secondary to kawasaki disease in young adults. *Circ J* 67**,** 362-365.

Newburger, J.W., Takahashi, M., Gerber, M.A., Gewitz, M.H., Tani, L.Y., Burns, J.C., Shulman, S.T., Bolger, A.F., Ferrieri, P., Baltimore, R.S., Wilson, W.R., Baddour, L.M., Levison, M.E., Pallasch, T.J., Falace, D.A., Taubert, K.A., Committee on Rheumatic Fever, E., Kawasaki, D., Council on Cardiovascular Disease in The, Y., American Heart, A., and American Academy Of, P. (2004). Diagnosis, treatment, and long-term management of Kawasaki disease: a statement for health professionals from the Committee on Rheumatic Fever, Endocarditis and Kawasaki Disease, Council on Cardiovascular Disease in the Young, American Heart Association. *Circulation* 110**,** 2747-2771.

Ogawa, S. (2011). [Overview of the JCS 2008 guidelines for diagnosis and management of cardiovascular sequelae in Kawasaki disease]. *Nihon Rinsho* 69 Suppl 9**,** 529-535.

Scanlon, P.J., Faxon, D.P., Audet, A.M., Carabello, B., Dehmer, G.J., Eagle, K.A., Legako, R.D., Leon, D.F., Murray, J.A., Nissen, S.E., Pepine, C.J., Watson, R.M., Ritchie, J.L., Gibbons, R.J., Cheitlin, M.D., Gardner, T.J., Garson, A., Jr., Russell, R.O., Jr., Ryan, T.J., and Smith, S.C., Jr. (1999). ACC/AHA guidelines for coronary angiography: executive summary and recommendations. A report of the American College of Cardiology/American Heart Association Task Force on Practice Guidelines (Committee on Coronary Angiography) developed in collaboration with the Society for Cardiac Angiography and Interventions. *Circulation* 99**,** 2345-2357.

Uehara R, N.Y., Yanagawa H. (2005). Epidemiology of Kawasaki disease in Japan. *JMAJ* 48**,** 183-193.

Yanagawa, H. (1999). Editorial comments: two adults of acute myocardial infarction after Kawasaki disease. *Shinzo* 31**,** 422-423.

Yanagawa, H., Nakamura, Y., Kawasaki, T., and Shigematsu, I. (1986). Nationwide epidemic of Kawasaki disease in Japan during winter of 1985-86. *Lancet* 2**,** 1138-1139.

Yoshikawa, J., Yanagihara, K., Owaki, T., Kato, H., Takagi, Y., Okumachi, F., Fukaya, T., Tomita, Y., and Baba, K. (1979). Cross-sectional echocardiographic diagnosis of coronary artery aneurysms in patients with the mucocutaneous lymph node syndrome. *Circulation* 59**,** 133-139.
